# Factor structure and internal consistency of the Clinical Assessment Interview for Negative Symptoms (CAINS) on a sample of persons with psychotic disorders in Bosnia and Herzegovina

**DOI:** 10.1007/s00702-021-02435-8

**Published:** 2021-12-08

**Authors:** Alma Džubur Kulenović, Eldina Smajic Mešević, Emina Ribić, Selman Repišti, Tamara Radojičić, Nikolina Jovanović

**Affiliations:** 1 Department for Psychiatry, Clinical Center of University of Sarajevo, Sarajevo, Bosnia and Herzegovina; 2 Department for Research and Development, Clinical Center University of Sarajevo, Sarajevo, Bosnia and Herzegovina; 3 Clinic for Psychiatry, Clinical Center of Montenegro, Podgorica, Montenegro; 4 Unit for Social and Community Psychiatry, WHO Collaborating Centre for Mental Health Services Development, Bart’s and London School of Medicine and Dentistry, Queen Mary University of London, London, UK

**Keywords:** Psychotic disorders, Negative symptoms, CAINS, Factor validity, Internal consistency

## Abstract

The assessment of negative symptoms is crucial for development of adequate therapeutic interventions. This is a challenging task due to complex clinical presentation and lack of reliable and valid instruments. This study examined the psychometric characteristics of the Clinical Assessment Interview for Negative Symptoms (CAINS). The sample consisted of 81 persons with schizophrenia or schizoaffective disorder recruited from two health institutions in the Sarajevo Canton: the Clinical Center of the University of Sarajevo and the Psychiatric Hospital of the Sarajevo Canton. The 13 CAINS items grouped into four factors (expression, motivation and satisfaction in the recreational domain, motivation and satisfaction with social relationships, motivation and satisfaction with job and education). The four-factor solution accounted for 87.83% of the variance of manifest items. The reliabilities of extracted factors were as follows: for motivation and satisfaction with social relationships *α* = 0.897, for motivation and satisfaction with job and education *α* = 0.961, for Motivation and satisfaction in the recreation domain *α* = 0.981, and for expression *α* = 0.938. The highest correlation between factors was found between Motivation and satisfaction with recreation and Motivation and satisfaction with social relationships. On the other hand, the lowest correlation was found between motivation and satisfaction with social relations and motivation and satisfaction with job and education. In conclusion, the study showed that the latent structure of CAINS is adequate, clearly interpretable, and consisted of four factors. The measure can be used for assessment of the negative symptoms in outpatients with psychosis in Bosnia and Herzegovina.

## Introduction

Schizophrenia is a chronic and severe mental disorder that affects more than 20 million people worldwide (GBD 2017 Disease and Injury Incidence and Prevalence Collaborators 2018). People with schizophrenia can often hear or see things that do not exist (hallucinations), have erroneous beliefs (delusions), or talk in an incoherent, intermittent, or incomprehensible way (disorganized speech). Those symptoms are defined as positive symptoms of schizophrenia.

Negative symptoms (blunting or flattening of affect, alogia/aprosody, avolition/apathy, anticipatory anhedonia, and asociality) are found in as many as 26% of patients with schizophrenia, and it is estimated that in up to 58% of outpatients they can occur at any time (Chue and Lalonde 2014). Negative symptoms contribute to decreased quality of life, increased functional disability, increased disease burden, and worse long-term outcomes, to a greater extent than positive symptoms (Horan et al. 2011). Despite their clinical relevance, therapeutic modalities did not have an unique approach to addressing negative symptoms for many years. To address this issue, European Psychiatry Association (EPA) guidance based on currently available evidence was recently published (Galderisi et al. 2021a). First of all, a switch to a second-generation antipsychotic should be considered for patients who are treated with a first-generation antipsychotic; adding antidepressant to antipsychotic treatment can be a valuable option, as well as social skills training and cognitive remediation for patients who also show cognitive impairment.

The assessment of negative symptoms is crucial for the development of adequate therapeutic interventions to improve patients' outcomes. However, this is a challenging task due to complex clinical presentation and lack of reliable and valid instruments. For example, negative symptoms are difficult to distinguish from symptoms of depression, side effects of medications, and secondary negative symptoms (symptoms resulting from long-term treatment with antipsychotics, chronic course of illness, and a long stay in the institution due to positive symptoms). Recent studies recognize significant limitations associated with current instruments used to assess negative symptoms (Chue and Lalonde 2014; Forbes et al. 2010).

National Institute of Mental Health (NIMH) Consensus Development Conference on Negative Symptoms presents a milestone for the development of second generation scales covering five negative symptom dimensions (alogia, social withdrawal, anhedonia, blunted affect and avolition) (Galderisi et al. 2021a). Negative symptoms rater scales developed before the conference are called first generation rater scales [Brief Psychiatric Rating Scale (BPRS), Scale for the Assessment of Negative Symptoms (SANS), Positive and Negative Syndrome Scale (PANSS), Schedule for Deficit Syndrome (SDS), and The Negative Symptoms Assessment (NSA)], and second generation rater scales were developed after this conference [The Brief Negative Symptom Scale (BNSS) and The Clinical Assessment Interview for Negative Symptoms (CAINS)].

EPA encourages clinicians to use second-generation scales, at least to complement first-generation ones (Galderisi et al. 2021b). Furthermore, EPA guidance recommends the evidence-based exclusion of several items included in first-generation scales from any negative symptoms summary or factor score to improve negative symptoms measurement in research and clinical settings. Self-rated instruments are suggested to further complement observer-rated scales.

CAINS is an empirically developed measure of negative symptoms (Forbes et al. 2010), developed to fulfil a major recommendation of the NIMH Consensus Development Conference on Negative Symptoms, using an iterative, empirical approach. CAINS include items that assess motivation, satisfaction, and expression of emotions (Kring et al. 2013).

The results indicate that CAINS is short but comprehensive and usable in a wide variety of research and clinical settings (Kring et al. 2013). CAINS was developed to compare the experience of satisfaction during assessment with expected satisfaction in the future (Horan et al. 2011). CAINS incorporated self-assessment components of expectations and fulfilment of satisfaction, where information is obtained through face-to-face interviews. Moreover, it also emphasizes emotional expression based on the items or behaviours observed during the interview. Affect flattening and alogia are classified as "expression" factors, while associativity, avolition and anhedonia are classified as "experience" factors (Horan et al. 2011).

CAINS is a 13-item interview instrument consisting of a motivation and satisfaction subscale (nine items) and an expression subscale (four items). Preliminary and subsequent large-scale clinical validation supports a two-factor structure for CAINS, namely, "expression" and "motivation/satisfaction" factors (Kring et al. 2013). This two-factor structure was found to be stable on the sample of respondents in Germany (Engel et al. 2014). CAINS is an important new addition to the tools available to assess negative symptoms in schizophrenia and other psychotic disorders, both theoretically and clinically. Theoretically, CAINS evaluates domains that portray the phenomenology of schizophrenia and the constructs of emotion, motivation, and processing. Clinically it provides better coverage of negative symptoms than some conventionally used instruments, e.g. distinguishes between expectations and experiences in the social, recreational, work and educational domains (Barch 2013). This instrument will serve as an important and valid tool for evaluating the negative symptoms of schizophrenia at various stages of the disease (Chan et al. 2015).

Aim of this study is to examine the psychometric characteristics of CAINS namely its factorial validity and internal consistency as type of reliability among mental health patients in Bosnia and Herzegovina.

## Methods

The study involved 81 people (44 women and 37 men) diagnosed with schizophrenia or schizoaffective disorder, whose age ranged from 18 to 65 years, whereas mean age was 41.70 (SD = 12.59). Participants were recruited in outpatient clinics at two locations in Sarajevo Canton (Clinical Center of the University of Sarajevo and Sarajevo Cantonal Psychiatric Hospital). The study included 16 clinicians/clusters (8 from each site) and each of them proposed five to eight patients from their caseload in accordance with inclusion criteria. A cluster-randomisation design was used to avoid potential contamination of the practice of clinicians when treating patients in intervention and control groups.

Patients met diagnostic criteria based on the structured clinical interview for DSM-IV disorders (SCID; First et al. 1997) The exclusion criteria were diagnosis of an organic brain disorder and presence of a serious cognitive deficit (patient unable to provide the information requested through the questionnaire). The fulfilment of the criteria was determined by clinical assessment. All participants signed an informed consent before the study began, and the study was previously approved by the ethics committees of the institutions where the study was conducted.

Translation of CAINS was done by two co-authors of this paper (Radojičić and Repišti, see Appendix 1). To assure linguistic validity, other co-author of the paper (Ribić) performed back translation of the scale.

### Procedure

Two co-authors of this paper are researchers who have completed the training required to credibly use the CAINS (ESM, ER). The training has been delivered by a lecturer in mental health care and principal investigator in multiple projects who holds a PhD in psychology. After the high level of interrater agreement had been established, the research with the CAINS was started. The assessment of patients/subjects was conducted in February and March 2019 at two locations: the Psychiatry Clinic of the Clinical Center of the University of Sarajevo and the Psychiatric Hospital of the Sarajevo Canton.

The CAINS is an interview-based assessment that includes the expression factor (vocal expression, gestures, facial expressions, and speech) and the motivation/satisfaction factor (recreation, social, and expected professional satisfaction and motivation). This is a semi-structured interview that follows the manual of the original authors. These items were evaluated based on the patient's reports of his/her motivation, his/her interests and feelings, as well as reports of actual engagement in relevant social, professional, and recreational activities. Satisfaction was rated in relation to the ability to enjoy the activities in the previous 7 days and the expected satisfaction in the next week. All items were rated on a scale of 0–4, where number 4 indicates a large deficit in functioning, while 0 indicates that there is no such deficit. Questions referred to the last seven days, except for the items related to expected satisfaction, which covered the following 7 days. The whole interview lasted an average of 30 min.

## Results

### Factor validity

To examine the factor structure of CAINS, a principal component analysis (PCA) was performed on sample *N* = 81. The a priori criterion was used for extraction, which would indicate that the number of latent factors was predetermined, in this case, based on the number of segments constituting the CAINS.

It was first examined whether the data collected and the relationships among them could be well captured and explained by a two-factor solution. Therefore, the first nine CAINS scores were expected to correspond to the factor of *Motivation and satisfaction*, while the remaining items should have been primarily saturated with the *Expression* factor. However, the principal component analysis in this case did not produce adequate results, since the *Expression* factor corresponded to 5th and 6th item as well. These two items, in fact, relate to motivation and satisfaction in the realm of *work and education*. On the other hand, the remaining items corresponded to the factor of *Motivation and satisfaction*.

The four-factor solution offered a better framework for interpreting the results. Two common preconditions for applying principal component analysis were satisfied: Kaiser–Meyer–Olkin sampling adequacy measure was KMO = 0.808, and Bartlett's test of sphericity resulted in a statistically significant value: *χ*^2^ = 1369, *df* = 78, *p* < 0.001. It should be noted that the factors were assumed to be correlated with each other; therefore, the *Promax* rotation was used. The extracted factors explained 87.83% of the variance of manifest variables.

Table 1 contains factor saturations of CAINS items by extracted factors, along with communalities (*h*^2^) and eigenvalues (*λ*).

**Table 1:** Principal component analysis results

Item	*h*^2^	I factor	II factor	III factor	IV factor
10. Facial expression	0.903	1.027	− 0.018	− 0.042	− 0.092
12. Gesture expression	0.835	0.935	− 0.049	− 0.070	0.074
11. Vocal prosody expression	0.883	0.894	− 0.026	0.057	0.050
13. Speech expression	0.759	0.754	0.054	− 0.033	0.173
9. Frequency of anticipated pleasant recreational activities—next week	0.963	− 0.078	1.023	− 0.034	0.042
8. Frequency of anticipated pleasant recreational activities—last week	0.958	0.020	0.996	− 0.045	− 0.008
7. Recreational activities—motivation	0.950	0.002	0.944	− 0.008	0.076
1. Family/partner relationships—motivation	0.715	− 0.221	− 0.263	0.997	0.227
3. Frequency of anticipated pleasant social activities—last week	0.918	0.036	0.224	0.819	− 0.086
4. Frequency of anticipated pleasant social activities—the following week	0.909	0.041	0.251	0.794	− 0.094
2. Close friendships/romantic relationships—motivation	0.723	0.384	− 0.051	0.648	− 0.102
5. Work and school activities—motivation	0.955	0.059	0.075	− 0.008	0.913
6. Frequency of anticipated pleasant work and school activities—next week	0.948	0.067	0.031	0.104	0.884
Eigenvalue (*λ*)		6.008	5.477	5.079	3.774

The first four items listed in Table 1 are good indicators of *Expression*, the first extracted factor. Items 7, 8, and 9 are indicators of the second factor, *Motivation and Satisfaction in the recreational domain*. Items 1, 2, 3 and 4 cover *Motivation and Satisfaction with social relationships*. Lastly, items 5 and 6 reflect *Motivation and Satisfaction with job and education*. *Expression* covers most of the variance in the item response, as the eigenvalue for this factor was the largest (*λ* = 6.008). The smallest part of the variance accounts for the last extracted factor, *Motivation and Satisfaction with job and education*. Eigenvalue for this factor is *λ* = 3.774. Communalities (*h*^2^) indicate how much variance of each of the items is explained by the extracted factors. Extracted factors explained the most variance in the case of item 9, *Frequency of anticipated pleasant recreational activities—next week*, *h*^2^ = 0.963), and least in the case of item 1, *Family/Partner relationships—motivation*, *h*^2^ = 0.715).

All correlation coefficients shown in Table 2 were statistically significant. Factors that correlate the most with each other are *Motivation and Satisfaction with recreation* and *Motivation and Satisfaction with social relationships* (*r*(77) = 0.561, *p* < 0.001). On the other hand, the lowest correlation was found between *Motivation and Satisfaction with social relationships* and *Motivation and Satisfaction with job and education* (*r*(77) = 0.286, *p* < 0.01).

**Table 2:** Intercorrelations of extracted factors

	Expression	Recreation	Social relationships	Work and education
Expression	1	0.539**	0.554**	0.526**
Recreation		1	0.561**	0.410**
Social relationships			1	0.286*

*p < 0.01; **p < 0.001

### Reliability

In addition to factor validity, internal consistency was also tested. The Cronbach's alpha (α) coefficient was used as an indicator of this type of reliability. Alpha coefficients were calculated for each part of the CAINS. All coefficients were high: *α* = 0.897 (*Motivation and Satisfaction with social relationships*), *α* = 0.961 (*Motivation and Satisfaction with job and education*), *α* = 0.981 (*Motivation and Satisfaction with recreation*), and *α* = 0.938 (*Expression*). Therefore, each of the CAINS segments had very good internal consistency. We have taken *α* = 0.7 as the confidence limit of this type, which is recommended by the rule of thumb.

The reliability of the whole instrument was also checked, which turned out to be very good (*α* = 0.933). In this regard, an item analysis was performed, the results of which are presented in Table 3.

**Table 3:** CAINS item analysis results

Item	Corrected item—total correlation	Cronbach’s alpha if item deleted
1. Family/partner relationships—motivation	0.465	0.935
2. Close friendships/romantic relationships—motivation	0.677	0.929
3. Frequency of anticipated pleasant social activities—last week	0.761	0.926
4. Frequency of anticipated pleasant social activities—next week	0.763	0.926
5. Work and school activities—motivation	0.689	0.929
6. Frequency of anticipated pleasant work and school activities—next week	0.633	0.932
7. Recreational activities—motivation	0.778	0.925
8. Frequency of anticipated pleasant recreational activities—last week	0.746	0.927
9. Frequency of anticipated pleasant activities—next week	0.721	0.927
10. Facial expression	0.718	0.928
11. Vocal prosody expression	0.779	0.926
12. Gesture expression	0.701	0.929
13. Speech expression	0.728	0.928

The highest corrected item-total correlation was obtained in the case of *Vocal expression* (*r*_it_ = 0.779). The next highest corrected item-total correlation is obtained for *Motivation for recreational activities* (*r*_it_ = 0.778). Thus, these two indicators correlate most with the total score on CAINS. On the other hand, *Family/Partner relationships—motivation* had the weakest (although still moderate) item-total correlation (*r*_*it*_ = 0.465). By excluding this item from CAINS, the reliability of the entire instrument would increase from *α* = 0.933 to *α* = 0.935. However, CAINS would lose out on its content validity. In the same manner, excluding some of the other items in CAINS would reduce its internal consistency reliability. Therefore, it can be stated that all items of this instrument should be retained.

## Discussion

This study sought to examine the validity of CAINS on a Bosnian sample of persons with psychotic disorders. The results of this study showed that the appropriate, clearly interpretable latent structure of CAINS included four factors and that each of the CAINS segments has very good reliability (more specifically, internal consistency).

The study also shows that the variability of 13 CAINS items is generated by the following four factors: *Expression, Motivation and satisfaction in the recreational domain, Motivation and satisfaction with social relationships,* and *Motivation and satisfaction with job and education.* Hence, the CAINS consists of four parts that correspond to the factors identified in our study. In other words, the factor structure of the Bosnian version of the CAINS reflects the four-section structure of this tool (the first three sections refer to the motivation and pleasure domain whereas the last one covers the expression domain).

These results are similar to the findings from a study conducted in Singapore, where there was also identified a four-factor structure of CAINS, and the extracted factors are the same as in the Bosnian sample (*Expression, Motivation and Satisfaction in the recreational domain, Motivation and Satisfaction with social relationships, Motivation and Satisfaction with job and education*) (Rekhi et al. 2019).

The factors that correlated to each other the most are *Motivation and satisfaction with recreation* and *Motivation and Satisfaction with social relationships.* On the other hand, the lowest correlation was found between *Motivation and Satisfaction with social relations* and *Motivation and Satisfaction with job and education.* Singapore’s study showed poor correlation between *Motivation and Satisfaction with recreation* and *Motivation and Satisfaction with social relationships* (*r* = 0.389, *p* < 0.05), while *Motivation and Satisfaction with job and education* and *Expression* do not correlate with any other factor. That can be explained by a general dissatisfaction with social conditions, poor socioeconomic situation, as well as lack of free time.

However, in most of the studies published so far, the two-factor structure of CAINS has been obtained. The two-factor structure (*Motivation/satisfaction* factor and *Expression* factor) was presented in the original version of the CAINS psychometric validation (Kring et al. 2013), and also in China (Chan et al. 2015), Spain (Valiente-Gomez et al. 2015), Korea (Jung et al. 2016), and Germany (Engel et al., 2014).

The structure of the negative symptoms and the relationship between its domains is not clearly defined. Prior to the development of the CAINS, factor-analysis studies examined the structure of negative symptoms using other first generation rater scales, such as PANSS, or SANS where negative symptoms were reported to be multidimensional, with two, three, or five factors (Blanchard and Cohen 2006; Kirkpatrick et al. 2006). Reduced expression and the combined anhedonia-asociality domain were considered to be the two most reliable domains (Blanchard and Cohen 2006; Peralta and Cuesta 1999). However, it was found that the reduced expression items could be considered as one factor when the ratings were based on observation during the interview, whereas the anhedonia–asocial domain items were matched because they were based on what the patient said about their activities and behaviour without thinking about what their intrinsic desires and motivations are (Azorin et al. 2014; Elis et al. 2013). Therefore, negative symptoms can have more than two basic domains.

The four-factor structure obtained in this study is in line with a recent publication on the structure of negative symptoms (Ahmed et al. 2019), which emphasized that two-factor models of negative symptoms might be premature, especially since most previous relevant studies have used exploratory factor analysis (EFA) alone and did not validate their research models using confirmatory factor analysis (CFA) (Ahmed et al. 2019).

The results of our study showed good internal consistency of both the CAINS as a whole and its four individual factors, which are consistent with the results of previously published studies (Kring et al. 2013; Engel et al. 2014; Chan et al. 2015; Valiente-Gomez et al. 2015; Jung et al. 2016; Rekhi et al. 2019).

This was the first validation study of the CAINS conducted in Bosnia and Herzegovina which is considered as the main strength of this research. Furthermore, clear inclusion and exclusion criteria were applied. This study has the potential to facilitate both research and clinical work in this area. As for its limitations, though the sample size was sufficient for PCA if we stick to the criterion of participants-items ratio being at least 5:1, the sample size was less than 100. The second limitation is that we did not control for extrapyramidal symptoms which could potentially mask the presentation of negative symptoms. Furthermore, there is a possibility that researchers failed to differentiate between depression, negative symptoms, and extra-pyramidal side effects while using the scale in certain more complex cases. Finally, interrater and test–retest reliability as well as convergent and divergent validity have not been checked, which could be regarded as a recommendation for further work on the psychometric validation of the CAINS in Bosnia and Herzegovina.

We hope that future studies will focus on checking the factor structure of CAINS-like instruments such as the Clinical Global Impression Scale (CGI), the Positive and Negative Syndrome Scale (PANSS), the Scale for the Assessment of Negative Symptoms (SANS), the 16-item Negative Symptoms Assessment (NSA-16), etc. We recommended using as many commonly used instruments of a high quality as possible to measure negative symptoms to check their latent structure and compare it with the DSM-5 categorisation. It is important to note that negative symptoms distinguished in the proposed way would be represented as dimensions (continua), as opposed to their usual, categorical conceptualisation.

## Conclusions

1. The relevant, clearly interpretable structure of CAINS in the Bosnian sample of persons with psychotic disorders is defined as a four-factor structure. The distinguishing factors (more precisely, dimensions or components), which correspond to the already defined segments of CAINS, are Expression, Motivation and Satisfaction with recreation, Motivation and Satisfaction with social relations and Motivation and Satisfaction with job and education.
2. The reliability (internal consistency) of CAINS as a whole, as well as its four segments individually, is very good.

## References

[CR1] Ahmed (2019). Cross-cultural validation of 5-factor structure of negative symptoms in schizophrenia. Schizophr Bull.

[CR2] Azorin JM, Belzeaux R, Adida M (2014). Negative symptoms in schizophrenia: where we have been and where we are heading. CNS Neurosci Ther.

[CR3] Barch DM (2013). The CAINS: theoretical and practical advances in the assessment of negative symptoms in schizophrenia. Am J Psychiatry.

[CR4] Blanchard JJ, Cohen AS (2006). The structure of negative symptoms within schizophrenia: implications for assessment. Schizophr Bull.

[CR6] Chan RCK, Shi C, Lui SSY, Ho KKY, Hung KSY, Lam JWS (2015). Validation of the Chinese version of the Clinical Assessment Interview for Negative Symptoms (CAINS): a preliminary report. Front Psychol.

[CR7] Chue P, Lalonde JK (2014). Addressing the unmet needs of patients with persistent negative symptoms of schizophrenia: emerging pharmacological treatment options. Neuropsychiatr Dis Treat.

[CR8] Elis O, Caponigro JM, Kring AM (2013). Psychosocial treatments for negative symptoms in schizophrenia: current practices and future directions. Clin Psychol Rev.

[CR9] Engel M, Fritzsche A, Lincoln TM (2014). Validation of the German version of the Clinical Assessment Interview for Negative Symptoms (CAINS). Psychiatry Res.

[CR01] First MB, Gibbon M, Spitzer RL, Williams JBW, Benjamin LS (1997). Structured clinical interview for DSM-IV axis II personality disorders, (SCID-II).

[CR10] Forbes C, Blanchard JJ, Bennett M, Horan WP, Kring A, Gur R (2010). Initial development and preliminary validation of a new negative symptom measure: the clinical assessment interview for negative symptoms (CAINS). Schizophr Res.

[CR11] Galderisi S, Kaiser S, Bitter I, Nordentoft M, Mucci A, Sabé M, Giordano GM, Nielsen MØ, Glenthøj LB, Pezzella P, Falkai P, Dollfus S, Gaebel W (2021). EPA guidance on treatment of negative symptoms in schizophrenia. Eur Psychiatry.

[CR12] Galderisi S, Mucci A, Dollfus S, Nordentoft M, Falkai P, Kaiser S, Giordano GM, Vandevelde A, Nielsen MØ, Glenthøj LB, Sabé M, Pezzella P, Bitter I, Gaebel W (2021). EPA guidance on assessment of negative symptoms in schizophrenia. Eur Psychiatry.

[CR13] GBD 2017 Disease and Injury Incidence and Prevalence Collaborators (2018). Global, regional, and national incidence, prevalence, and years lived with disability for 354 diseases and injuries for 195 countries and territories, 1990–2017: a systematic analysis for the Global Burden of Disease Study 2017. Lancet.

[CR14] Horan WP, Kring AM, Gur RE, Reise SP, Blanchard JJ (2011). Development and psychometric validation of the clinical assessment interview for negative symptoms (CAINS). Schizophr Res.

[CR16] Jung SI, Woo J, Kim YT, Kwak SG (2016). Validation of the Korean-Version of the Clinical Assessment Interview for Negative Symptoms of Schizophrenia (CAINS). J Korean Med Sci.

[CR17] Kirkpatrick B, Fenton WS, Carpenter WT, Marder SR (2006). The NIMH-MATRICS consensus statement on negative symptoms. Schizophr Bull.

[CR18] Kring AM, Gur RE, Blanchard JJ, Horan WP, Reise SP (2013). The Clinical Assessment Interview for Negative Symptoms (CAINS): Final Development and Validation. Am J Psychiatry.

[CR19] Peralta V, Cuesta MJ (1999). Dimensional structure of psychotic symptoms: an itemlevel analysis of SAPS and SANS symptoms in psychotic disorders. Schizophr Res.

[CR20] Rekhi G, Ang MS, Yuen CKY, Ng WY, Lee J (2019). Assessing negative symptoms in schizophrenia: validity of the clinical assessment interview for negative symptoms in Singapore. Schizophr Res.

[CR21] Valiente-Gomez A, Mezguida G, Romaguera A, Vilarderbo I, Andres H, Granados B, Larrubi J, Pomarol-Clotet E, McKenna PJ, Sarro S, Bernardo M (2015). Validation of the Spanish version of the Clinical Assessment for Negative Symptoms (CAINS). Schizophr Res.

